# Biomedical and Microbiological Applications of Bio-Based Porous Materials: A Review

**DOI:** 10.3390/polym9050160

**Published:** 2017-04-29

**Authors:** T. M. S. Udenni Gunathilake, Yern Chee Ching, Kuan Yong Ching, Cheng Hock Chuah, Luqman Chuah Abdullah

**Affiliations:** 1 Department of Chemical Engineering, Faculty of Engineering, University of Malaya, Kuala Lumpur 50603, Malaysia; sampatht@siswa.edu.my; 2 University of Reading Malaysia, Persiaran Graduan, Kota Ilmu, Educity, Iskandar Puteri Johor 79200, Malaysia; K.Y.Ching@reading.edu.my; 3 Department of Chemistry, Faculty of Science, University of Malaya, Kuala Lumpur 50603, Malaysia; chchuah@um.edu.my; 4 Department of Chemical Engineering, Faculty of Engineering, Universiti Putra Malaysia, Serdang 43400, Malaysia; chuah@upm.edu.my

**Keywords:** biomaterial, biocompatibility, porosity

## Abstract

Extensive employment of biomaterials in the areas of biomedical and microbiological applications is considered to be of prime importance. As expected, oil based polymer materials were gradually replaced by natural or synthetic biopolymers due to their well-known intrinsic characteristics such as biodegradability, non-toxicity and biocompatibility. Literature on this subject was found to be expanding, especially in the areas of biomedical and microbiological applications. Introduction of porosity into a biomaterial broadens the scope of applications. In addition, increased porosity can have a beneficial effect for the applications which exploit their exceptional ability of loading, retaining and releasing of fluids. Different applications require a unique set of pore characteristics in the biopolymer matrix. Various pore morphologies have different characteristics and contribute different performances to the biopolymer matrix. Fabrication methods for bio-based porous materials more related to the choice of material. By choosing the appropriate combination of fabrication technique and biomaterial employment, one can obtain tunable pore characteristic to fulfill the requirements of desired application. In our previous review, we described the literature related to biopolymers and fabrication techniques of porous materials. This paper we will focus on the biomedical and microbiological applications of bio-based porous materials.

## 1. Introduction

In the competitive world, synthetic polymers in which the desired properties can be easily modified according to the needs are generally used as substitutes for biopolymers. Degree of branching, molecular weight, chemical substituents, and main chain sites can be more readily changed and organized in synthetic polymers than in most biomaterials [1,2]. Some biomaterials show property variations resulting from different environmental and agronomic factors, such as weather and other complicated considerations. These limitations render biopolymers relatively unattractive at the commercial level [3].

At present, the socio-economic situation has changed to widen the applications of biopolymers. Issues on oil supply, oil price hike, and sustainability have directed scientists to focus on alternative and sustainable resources [4]. In addition, natural materials are environmental friendly because of their biodegradability, non-toxicity, and low disposal costs [5]. Moreover, the use of natural materials such as starch for fillers in composite materials is cheaper compared with the use of synthetic polymer matrices [6]. These biopolymers consist of polysaccharides, such as starch, cellulose, chitin/chitosan, alginate, and polysaccharides (wool, gelatin, silk, and collagen) synthesized from bacterial, fungal, and animal proteins [7,8].

As defined by Williams [9], the biocompatibility is a characteristic of a system and not a material. Furthermore, biomaterial is a substance that has been engineered to interact with biological systems in controlled manner [9,10]. Therefore, it is described that biomaterial as biocompatible system when discussed about its biocompatibility characteristics.

Given their uses as biocompatible systems, these biomaterials are superior over conventional polymer materials in various applications [11]. As biocompatible systems, these biomaterials may interact with different biological systems in different ways such as tissue processes involved in wound healing, the endothelium in contact with intravascular devices, the target cells in gene therapy and the stem cells in bioreactors. In many products of medical technology, more than one biomaterials may involve and the interactions between these materials may play some role in biocompatibility [9].

Biomaterials are widely used in many applications because of their high specific surface area, high specific strength, and low relative density [12,13,14,15]. Modern technologies provide powerful tools to fabricate the porous structures of biomaterials and to enhance the natural biosynthetic systems for targeted applications. Porous architectures have been fabricated using different techniques, such as solvent casting, particulate leaching, gas foaming, phase separation, electrospinning, porogen leaching, fiber mesh, rapid prototyping, and freeze drying, to meet the requirements of different applications [16].

Porous biopolymer matrices are used for biomedical applications such as tissue engineering, control drug delivery systems and wound healing [17]. It is also a popular candidate for microbiological applications such as encapsulation of microorganism in fermentative industry, in the production of probiotic food, biosorption and bioremediation of chemicals and as antimicrobial food packaging material [18]. In tissue engineering, scaffolds provide a framework for cell attachment, migration, differentiation, and induction of new tissue shape formation [19]. Aside from biodegradability, biocompatibility and mechanical properties, porosity is also an important consideration when selecting a desirable scaffold for tissue engineering [20]. Considering the stable and uniform porous structure, tunable pore sizes, increased surface area and surface properties of porous materials, many studies have exerted considerable efforts to develop porous biopolymers as controlled drug delivery matrices [21]. The porosity of the biopolymer matrix is important for wound healing applications because it allows cell filtration and provides high permeability for the diffusion of oxygen and nutrients [22]. Several biopolymers are available for microbial encapsulation in porous matrices to hold the microorganisms for water purification, biomolecule production, and ethanol production. Encapsulation of microorganisms in porous matrix provide several advantages such as resistance to environmental stressor and enhance the viability of microorganisms, promote controlled release and optimize delivery of microorganisms (probiotic) to the site of action [23]. Bio-based porous materials have been used to incorporate antimicrobial agents to food packaging materials for preservation of food. It provides an effective way to control spoilage microorganisms and food-borne pathogens thus enhance food safety and decrease product spoilage [23].

This paper presents a review regarding the biomedical and microbiological applications of bio-based porous materials and the details of intricate features of the microarchitecture of porous matrices, such as porosity and pore morphology, fabricated using various methods.

## 2. Biomedical Applications

### 2.1. Porous Scaffold for Tissue Engineering

Disease, injury, or trauma can damage tissues, and treatments are needed to facilitate the repair, replacement, or regeneration of damaged tissues in the human body. Typical treatments include tissue transplantation from one site of the body to another or from one individual to another. Major problems associated with these techniques are painful operation, high cost, risk of refusal by the patient’s immune system, and risk of introducing infection or disease from the donor to the patient. Tissue engineering aims to regenerate damaged tissues without replacing them by combining body cells on a highly porous scaffold biomaterial, which acts as a template for tissue regeneration. These templates should not only provide biodegradability, biocompatibility, and other mechanical features but also high porosity and interconnected pore structure to facilitate the satisfactory diffusion of nutrients to cells within the template and to the extracellular medium formed by these cells [24]. Biodegradability is often an essential feature of a biomaterial used in tissue engineering since it acts as a temporary template for tissue forming in place until the natural extracellular matrix has sufficiently developed. Beyond that point, the scaffold should degrade into nontoxic end products which are capable of being disposed of by the body leaving only the newly formed tissue.

**Figure 1:**
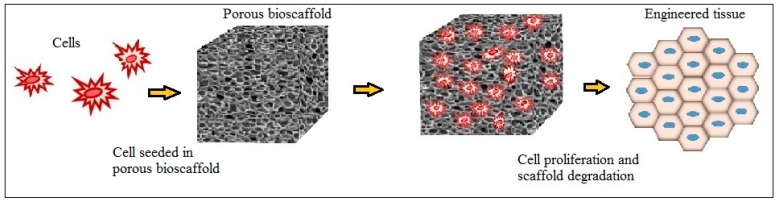
Schematic representation of tissue regeneration using porous bioscaffold.

A wide variety of cell seeding techniques have been explored to improve cell seeding efficiency and uniform distribution [25,26,27,28,29,30]. The simplest of which involves placing the desired amount of cell suspension on the top of the scaffold and letting the liquid slowly drip into the porous structure of the scaffold. After initial seeding, the scaffold is removed from culture plate and placed in the site of implantation of the body. As the cells grow, migrate, and multiply, the scaffolding degrades naturally in the body leaving newly formed tissue. Figure 1 represents the process of tissue formation in the cell-implanted bioscaffold.

The surface area-to-volume ratio of these porous matrices depends on the density and average diameter of the pores. Given that the diameter of cells dictates the minimum pore size depending on the intended applications, the pore size of the scaffold must be carefully controlled. Table 1 shows the optimal pore size for cell infiltration reported by previous researchers [25,26,27,28,29,30]. When the pores are too small, seeding cells in the middle of the scaffold and feeding the inner parts of the scaffolds are limited. Large pores improve the stability of the scaffold and help provide physical support for the seeded cells [31].

Scaffolds with a desired porosity, pore size and interconnectivity are required by various tissue engineering applications because the pore morphology strongly affects the cell growth. Ideal pore sizes vary for different cells and tissues. Fabrication methods are closely related to pore size and structure. Pore size, pore interconnectivity and porosity can be controlled by varying the process parameters of fabrication method and material composition of scaffold matrix.

#### 2.1.1. Porous Scaffold by Thermally Induced Phase Separation

Thermally induced phase separation is a well-known, simple and versatile fabrication method for the preparation of porous scaffolds. In this technique, a polymer solution is prepared at high temperature and phase separation is achieved by cooling down the homogenous solution. Subsequent freeze-drying of the phase-separated polymer solution produces porous structures as a result of solvent removal. Ma et al. [32] fabricated porous scaffolds from poly(l-lactic acid) through thermally induced phase-separation for the cultivation of small-diameter blood vessels. It was observed that the porosity reduced from 95% to 90% and the average pore size decreased from 120–150 µm to 80–120 µm when the polymer concentration was increased from 2.5% to 10%. The pore size greatly decreased from 115–140 µm to 20–40 µm with the decrease in phase-separation temperature from −20 to −196 °C. Pavia et al. [33] developed chitosan/poly(dl-lactide-*co*-glycolide) composites by thermally induced phase separation for applications in tissue engineering. Composites were frozen at −78 °C for 3 or 6 h. The solvent was then extracted by a freeze drying. It was found that more uniform and larger pores were formed inside the composite than in the surface. This was because of temperature gradient of the cooling rate of the freezing process. In addition, faster cooling rates tend to produce smaller crystals because there is not sufficient time for large particles to form. As a result, small pore sizes were obtained at faster cooling rates, because the cooling rate was inversely related to the crystal size obtained [34].

#### 2.1.2. Porous Scaffold by Freeze-Drying/Lyophilization

Freeze-drying/lyophilization is another simple and versatile technique that can be applied for fabrication of broad range of biomaterial scaffolds. It involves freezing the material and then reducing the surrounding pressure to allow the frozen water in the material to sublimate. The porous structures are created by the ice crystals that sublimate, leaving gaps or pores in their place. Li et al. [35] used lyophilization to prepare porous chitosan scaffolds for cultivation of rat hepatocytes. Scaffolds with a porosity of 90% and mean pore sizes ranging from 50 to 200 µm were obtained using this method. The processing conditions were 4 °C for 6 h for gellation, frozen at −28 °C, and then lyophilized in a freeze-dryer followed by rehydration step. Hydroxyapatite and gelatin composite scaffolds were prepared using solvent casting combined with freeze drying. The molds were frozen at −70 °C and then dried in a commercial freeze-dryer for 6 h for solvent removal. Results showed that the prepared scaffold had an open, interconnected porous structure with a pore size of 80–400 µm [36]. Chitosan scaffolds were prepared by cross-linking with glutaraldehyde for cultivation of human dermal fibroblasts. The porosity and pore size of the scaffolds were controlled by varying the freezing rate to form ice crystals of varying sizes. With this technique, the pore size of the scaffold was 40–140 µm, and the average porosity was about 93% ± 12.57% [37].

#### 2.1.3. Porous Scaffold by Supercritical Fluid Processing

The use of supercritical CO_2_ for the fabrication of porous scaffold has attracted interests in recent years due to the absence of using organic solvents. Pore structure and porosity of the scaffold can be effectively controlled by varying the saturation pressure, temperature, and processing time of this method. Porous scaffolds were prepared using composite biomaterials (poly(lactic-*co*-glycolic acid)/hyaluronic acid/collagen) and fabricated with supercritical CO_2_ for osteoblast cell culture. A porosity of 88.9% and a pore size of 205.7 µm were obtained when the scaffold was fabricated at 18 MPa pressure and 45 °C for 45 min [38]. Teng et al. [39] had reported that the porosities of poly (d,l) lactic acid/hydroxyapatite scaffold decreased with increasing the CO_2_ saturation pressures. It was observed that the porosity decreased from 92% ± 2% to 47% ± 2% when the pressure was increased from 10 to 14 MPa. It was also found that the porosity of the scaffold increased when the saturation time increased from 15 to 60 min. In addition, the porosity of the composites at 40 °C was slightly higher than that of composites formed at 35 °C under the same saturation time.

Table 2 lists the summary of pore characteristics of biopolymer scaffolds prepared from different fabrication methods for culturing different types of cells [32,35,36,37,38].

### 2.2. Porous Carriers for Drug Delivery

**Figure 2:**
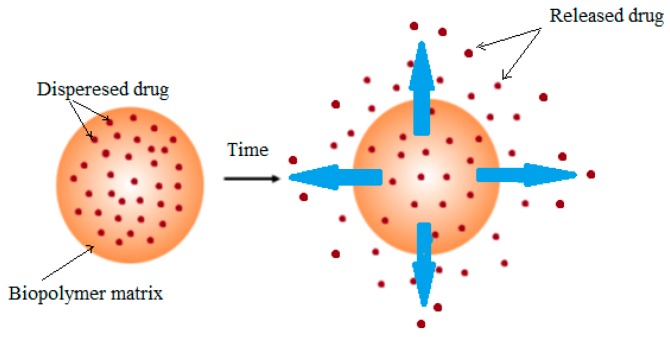
Drug diffusion from the biopolymer matrix containing dispersed drug.

In controlled drug delivery systems, an active therapeutic agent is integrated into a polymeric matrix to release the drug from the material in a predetermined manner. Depending on the drug delivery design and the application, the drug release time may be a few hours or a few months to several years [40]. Figure 2 illustrates the drug diffusion from the biopolymer matrix containing dispersed drug at a specific time.

Hydrogels are cross-linked polymer matrices with several hydrophilic groups or domains. Although these materials are hydrophilic, the presence of chemical or physical bonds between polymer chains prevents the dissolution of hydrogel in water. Binding of polymer chains is achieved either by noncovalent physical associations and physical entanglements or by covalent cross-linkages [41].

Drugs are incorporated into hydrogel in two ways. One is through post-loading, wherein a drug is loaded after hydrogel networks are formed. If the hydrogel system is inert, diffusion is the major driving force for drug uptake and release. In the presence of drug-binding ligands in the hydrogel, drug-polymer interaction and drug diffusion must both be considered in any model description of release. The other is through in situ loading, wherein drugs or drug-polymer conjugates are introduced to polymer precursor solution. In this method, hydrogel formation and drug encapsulation occur simultaneously. In such cases, drug release depends on hydrogel swelling, diffusion, reversible drug-polymer interactions, or degradation of labile covalent bonds [42].

When the pore size of a hydrogel is greater than the molecular size of the drug, the diffusion coefficient can be related to the porosity and tortuosity of the hydrogel. For hydrogels with pore sizes closer to the drug molecular size or nonporous hydrogels, drug diffusion coefficients are decreased because of the steric hindrance of polymer chains. In such cases, the average free volume for drug molecules is decreased and the hydrodynamic drag toward the drug is increased, causing increased drug diffusion path length compared with hydrogels with pore sizes much larger than that of the encapsulated drug [42].

A wide range of biopolymers have been investigated as drug carriers. Porous biomaterials as drug delivery carriers have been designed using natural polymers, such as chitosan, due to their well-documented biodegradability, nontoxicity and biocompatibility. Phaechamud and Charoenteeraboon [43] had developed chitosan sponge containing doxycycline hyclate using freeze drying, and the drug release and sustainable antibacterial activity of this material were studied in the presence of a high concentration of chitosan in the hydrogel. The pore density of the sponge prepared with 10% (*w*/*w*) chitosan solution was higher than that of the sponge prepared with 4% and 7% (*w*/*w*) chitosan solutions; the shape and volume of the pores were consistent. In the presence of a high concentration of chitosan, the pores were well interconnected and the pore diameter was about 80–130 µm. The high pore surface area allowed a large amount of the drug to be loaded into the matrix. The pore volume of the chitosan sponge diminished after cross-linking with glutaraldehyde solution. However, doxycycline hyclate could be effectively loaded to cross-linked chitosan sponge, and the drug release from the cross-linked chitosan sponge was higher than that from the noncross-linked chitosan sponge. Mirzaei B et al. [44] had investigated the properties of glutaraldehyde-cross-linked chitosan hydrogel with varying cross-linking concentrations for drug delivery system of amoxicillin trihydrate. The pore size increased from 100 to 500 µm with increasing cross-linking agent from 1:0.068 to 1:0.30. Hydrogel with 20 mol % cross-linker showed the best swelling behavior for drug release [44].

#### Microporous and Superporous Matrices for Drug Delivery

Porous materials are highly attractive as controlled drug delivery matrices because of their controllable pore size and porous structure. Microporous and superporous structures of polymer matrices control the passage between the external and internal surfaces of a solid, allowing materials to pass in or out of the solid [18]. Microporous-structured poly-lactic acid scaffolds were prepared using a robotic dispensing technique and room-temperature ionic liquid for drug loading and delivery studies of ampicillin and cytochrome C. Macroporous channels with controlled pore configuration were obtained by a robotic dispensing technique. Room-temperature ionic liquid created the bicontinuous interpenetrating network in the macroporous channels formed by robotic dispensing. The average pore size was 2.43 µm, and the microporosity was ~70%. The microporous scaffolds showed greater drug loading capacity (4–5 times increase in ampicillin and 9–10 times increase in cytochrome C) compared with the nonmicroporous scaffolds. The release of ampicillin from microporous scaffolds was initially faster and then slowed down, showing continual release over a month. Cytochrome C exhibited a sustainable release over a month [45]. Microporous hydroxyapatite/chitosan composite beads were prepared by ionic crosslinking to be used as a system of sustained drug delivery to bone. Beads were formed to encapsulate tetracycline hydrochloride by ionic crosslinking using tripolyphosphate and freeze drying. Open pore channels and an interconnected framework were obtained, and most of these pores were of irregular shape. The average pore diameter was about 45 ± 17 µm. The pore size decreased with increasing hydroxyapatite content. In addition, small pores facilitated higher absorption of drug and sustained release compared with large pores [46]. Carboxymethylcellulose-based microporous hydrogels were prepared by crosslinking with 1,3-diaminepropane. Microporous structure with different pore sizes (14, 30 and 40 µm) was obtained and drug release kinetics was analyzed for ibuprofen-lysin. It was reported that the drug release rate extended from 24 h to 7 days with increasing the pore size of the microporous hydrogel from 14 µm to 30–40 µm. This was mostly due to the more internal distribution of the drug in the largest pore dimension hydrogels [47].

Superporous hydrogels consist of hydrophilic polymers and show high swelling ratios and rapid swelling properties due to the presence of interconnected microscopic pores. Bioadhesive superporous hydrogel composite particles were prepared from hydroxypropyl-methylcellulose and chitosan biopolymers using gas blowing for intestinal drug delivery. Composite particles with a pore size of 100–1000 µm and a porosity of 47.11% ± 1.80% were obtained for drug (metoprolol succinate) delivery studies. The particles showed more than 80% drug loading and drug release up to 10 h [48]. Chitosan/poly(vinyl alcohol) interpenetrating polymer network type superporous hydrogel was prepared using gas foaming method for the drug delivery of rosiglitazone maleate [49,50]. Superporous hydrogels showed very fast swelling rate which resulted in high swelling ratio. It showed a rapid release of drug and reached to equilibrium state around 100 min. In addition, release profile showed similar pattern with the swelling properties of these superporous hydrogel [49]. The interconnected pores and capillary channels are the typical features of superporous hydrogel network. Owing to its porous structure, it exhibits faster swelling rate as well as larger equilibrium swelling ratio. Udeni Gunathilake et al. [51] reported the development of superporous chitosan hydrogel reinforced with nanocellulose for the enhancement of bioavailability of curcumin. Highly interconnected and large pore structures were fabricated using carbon dioxide gas foaming method. It was observed that the drug loading efficiency and amount of drug release increased in the hydrogel fabricated using gas foam method when compared with the hydrogels formed at atmospheric condition. Table 3 lists the summary of pore characteristics of different drug delivery matrices prepared from various fabrication methods [43,44,45,46,48,49,50,51].

### 2.3. Wound Healing Material

Wound dressings are commonly used in wound healing. An ideal wound dressing material should allow gaseous exchange, absorb exudates and toxic compounds, maintain high humidity at the applied surface, provide thermal insulation, protect the wound from bacterial contamination, be nontoxic, be removed easily, be easily handled without any damages, be sterilizable, and be free from leaving foreign particles in the wound. Biodegradable wound dressings can be used to treat wounds that are difficult to remove [52].

Depending on the required property, the hydrophilicity, swelling ratio and porosity, and degradation of a wound dressing material are controlled using bioactive hydrogels to regulate the rate of fluid passage from the wound, enhance the diffusion of encapsulated drug, and release the by-products into the wound and help in tissue regeneration, respectively [53]. Water absorption behavior is an important characteristic of wound dressing materials. The absorption of excessive fluid in wound surface is critical to wound healing. Water uptake is related to the capillary capacity of the material, which generally depends on the size and amount of pores. However, the suction capacity does not depend completely on porosity. The open porosity (accessible by the liquid) and pore tortuosity of the solid also affect the capillary capacity of the material [54].

Different bioactive hydrogels, such as those based on chitosan, collagen, hyaluronic acid, alginate, or elastin biopolymers, can be used to control the properties of wound dressing materials. Alginate-based dressings can be used to treat dry wounds after treatment with saline. They show high swelling ratios and can absorb large exudate volumes in wounds. As the main structural protein in connective tissues, collagen provides extracellular matrix structures for wound dressing materials [53].

Chitin and chitosan stimulate cell adhesion and proliferation, as well as help in the organization of the extracellular matrix. The antibacterial and fungicidal properties of both polymers also render them attractive for wound healing applications. Hyaluronan is another main component of the extracellular matrix used for chronic wound treatment, and elastin is a protein present in connective tissues that demonstrates load-bearing and stretchable properties. Elastin participates in extracellular matrix production, cell migration, and protease synthesis [53,55].

The antibacterial activity is also an important factor on selecting a wound dressing for wound healing. Straccia et al. [56] developed alginate hydrogels coated with chitosan for wound dressing and studied the antibacterial activity on *Escherichia coli.* Antibacterial activity was carried out using solid agar medium contact method. Results showed that presence of microbial inhibition zone around the contact area in the case of coated hydrogels and completely absent in uncoated hydrogels [52].

Mechanical properties are the most important character in hydrogel properties, which prevent physical damage to the wound and to support easy handling and storage. The mechanical and functional properties of collagen and fibrin hydrogels were characterized for wound healing applications. The pore sizes of 2.84 and 1.69 µm and the void ratios of 80.15% and 71.46% were obtained for collagen and fibrin hydrogels, respectively. In addition, fibrin hydrogel showed lower permeability and greater shear modulus compared with collagen hydrogel [57]. Kim et al. [58] prepared poly(vinyl alcohol)/alginate hydrogel containing nitrofurazone for wound dressing purposes. They have used the freeze-thawing method to crosslink poly(vinyl alcohol)/sodium alginate blended polymer. It was observed that the mechanical properties of poly(vinyl alcohol)/sodium alginate hydrogel film increased with increasing the sodium alginate contents. El Salmawi [59] reported the preparation of poly(vinyl alcohol)/chitosan wound dressing hydrogel using different doses of γ-radiation to induce crosslinking. Results referred that the mechanical properties of the hydrogels blend increased with increasing poly(vinyl alcohol) concentration.

## 3. Microbiological Applications

### 3.1. Encapsulation of Microorganisms in Food Industry

For successful immobilization of microorganisms, a matrix must exhibit high chemical and biological stability, mechanical strength, large surface, porosity and appropriate permeability to diffusion and transport of oxygen, essential nutrients, metabolic waste and secretory products.

Brewing and winemaking industry are mainly based on the microorganisms for their alcoholic fermentation process. Instead of using free cells, fermentation process is facilitated by immobilizing the yeast cells on several organic materials such as polysaccharides (calcium alginate, carrageenan, pectin), poly(vinyl alcohol), modified polystyrene and modified polyethylene. It is reported that in winemaking process, cell immobilization on biomaterials such as alginate, cellulose, carrageenan, agar, pectine, chitosan and gelatine contributes inhibiting the toxic influence of produced ethanol on microorganisms. Immobilization of microorganisms improve the condition of the process as well as on the properties of the product such as quality of their flavor [60].

There are two methods of entrapment of yeast to the porous matrices. In the first method cells are allowed to diffuse in to the porous matrix. The movement of grown cells is hindered by other cells and the matrix. In the second method, porous matrix is formed in situ around the cells. Natural polymeric materials such as Ca-alginate, α-carrageenan and agar are being used to form the hydrogel beads with this method. These polymeric hydrogel beads have less attraction in fermentation industry due to several drawbacks such as chemical and physical instability, limited diffusion of nutrients, oxygen and metabolites and high cell population in gel beads [61,62].

Traditional beer fermentation technology with freely suspended yeast cells takes about 7 days for the production of beer with a subsequent maturation stage of several weeks. Higher fermentation temperature with selected specific yeast strain takes about 12–15 days to finish the production of beer. Immobilized cell technology is able to produce beer within 1–2 days. The main difficulty is to obtain the correct balance of sensory compounds to give the acceptable flavor profile within short period of time [63]. Another problem related to this method is leakage of yeast cells from the matrix to the medium. Some studies demonstrated that this problem can be overcome by creating a barrier membrane to the gel matrix. This can be achieved by the addition of an extra polymer [64].

Microorganisms produce aldehydes, ketones and acids by the partial degradation of sugar, alcohol and aldehyde by using their enzymes. *Gluconobacter oxydans* is a very small size microorganism leading to difficulties in the reuse or recycling of the cells for large-scale production processes. By immobilizing bacteria to porous chitosan sponge, 92% activity recovery and 74% reusability were obtained [65]. Also, no cell loss was observed in this process. Morphological studies showed that cells were attached to the surface of the pores (100–400 μm) and the activity recovery increased with increasing the porosity [65].

Different matrices can be used to immobilize microorganisms to improve the productivity of vinegar/acetic acid. *Acetobacter aceti* bacterial cells were immobilized in calcium alginate gel beads by fluidized bed type column reactors. The study revealed that both of the concentration and productivity of the acetic acid in this immobilized cell systems were two fold greater than those in the free cells systems [66]. Fumi et al. [67] reported that the oxygen uptake rate decreased with increasing the alginate concentration of calcium alginate beads used for immobilization of acetobacter in vinegar production. This may be due to the fact that the porosity of the gel beads decreased with increasing alginate concentration. Sun and Furusaki [68] found that the productivity of acetic acid increased at the lower dilution rate for the system with larger gels. However, the productivity decreased with the increase of the dilution rate for larger gels. This decrease was due to the significant decrease in the suspended cell population, which caused the cell catalyzed reaction rate to decrease. Studies [69] were also carried out to investigate the effect of pH and temperature on immobilized bacteria used for production of acetic acid. The studies showed that there was no significant alteration for the production of acetic acid on changing the temperature and pH for immobilized bacteria [69].

### 3.2. Encapsulation of Probiotic Bacteria

Probiotics are live microorganisms which confer health benefits to the host by maintaining or improving their intestinal microflora [70]. Today there are many probiotic-based health products available in the market in the form of fermented dairy products as well as dietary supplements. Probiotic bacteria have to survive during the time from processing to consumption of a food product. They need to be protected from processing conditions (temperature, oxidation, etc.), storage conditions (moisture, oxygen, and temperature), high acidic conditions in the stomach and bile salts in the small intestine. The encapsulation techniques are developed to enhance the viability of these microorganisms in food products as well as in the gastrointestinal tract [71]. The matrix used for the encapsulation should have (a) chemical, physical, and biological stability during the production process (b) sufficient mechanical strength (c) biocompatibility (d) biodegradability (e) higher loading capacity (f) physical characteristics such as porosity, compression and swelling [72].

Alginate matrices are attractive candidates for the encapsulation of probiotic microorganisms due to their versatility, biocompatibility and non-toxicity. Probiotic strains namely *Staphylococcus succinus* and *Enterococcus fecium* co-encapsulated with complementary prebiotics on alginate matrix. They used an oligosaccharide-rich carbohydrate source as such in encapsulation, and was found to have an improved survival rate of probiotic strains. Results revealed that alginate microspheres were more densely loaded with probiotic bacteria. Co-encapsulated cells showed approximately 88.75%–98.75% of survivability when exposed to simulated gastric environment [73]. *Lactobacillus casei* and *Bifidobacterium bifidum* were encapsulated using calcium alginate-gelatinized starch with chitosan coating. The results showed that the survival of probiotic bacteria within encapsulated matrix increased significantly in simulated gastro-intestinal condition. Gelatinized starch with chitosan coating reduced the porosity and decreased the cell leakage of encapsulated probiotics. The results proved that the reduction of pore size and distribution of gastric juice in double coated sodium alginate membrane lead to limitation of interaction between cells with the gastric juice [74]. Moreover, cellulose acetate phthalate (a cellulose derivative polymer) is physiologically inert and can be used for the encapsulation of probiotic bacteria for the delivery in the intestinal tract. *Bifidobacterium pseudolongum* was encapsulated in cellulose acetate phthalate using phase separation-coacervation technique. *Bifidobacterium pseudolongum* is used for the replacement therapy for several bacterial-induced gastrointestinal disorders. Results revealed that the microencapsulated *B. pseudolongum* survived in the simulated gastric environment in larger numbers than non-encapsulated *B. pseudolongum.* Therefore, microencapsulation of *B. pseudolongum* in cellulose acetate phthalate seem to offer an effective way of delivering large numbers of viable bacterial cells to the gastrointestinal tract [75].

### 3.3. Antimicrobial Food Packaging

Traditionally antimicrobial agents are mixed during the food formulating stage to prevent the growth of microorganisms and to extend the shelf life of the food. Reactions and the interactions of antimicrobial agents with the food system cause neutralization and decrease or cease the protective ability. Also, the antimicrobial agent cannot penetrate to the surface of the food where the food spoilages are more intensive. To overcome these limitations, antimicrobial packaging materials were developed with controlled releasing rates [76]. Development of antimicrobial packaging material by using biopolymers is a great attempt towards attaining sustainability in food packaging applications [77].

**Figure 3:**
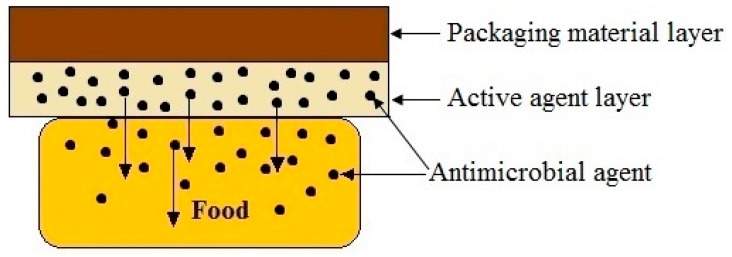
Releasing of antimicrobial agent from food contacting layer of multilayer packaging material.

There are several approaches of incorporation of antimicrobial agent to the food packing material. One is mixing of the antimicrobial agent in the extruder when the film is produced. This is a poor cost effective method. It is due to the fact that antimicrobial agent which is not exposed to the surface of the film will not involve with antimicrobial activity. To overcome this drawback, antimicrobial agent is introduced to the food contacting layer of multilayer packaging material [15]. Figure 3 illustrates the releasing of antimicrobial agent from food contacting layer of multilayer packaging material.

Cellulose-based materials are widely used to produce antimicrobial food packaging materials due to their edibility, biocompatibility, barrier properties, non-toxicity and low cost. Gemili et al. [78] developed cellulose acetate (CA) films with different morphological features to study the release rates of low molecular weight natural antioxidants (l-ascorbic acid and l-tyrosine). It was found that porosity and pore size of the film was decreased with increasing the cellulose acetate concentration. This has caused a reduction of diffusion rates of both antioxidants through the film. Highest antioxidant activity was observed with highly porous l-tyrosine containing films. When decreasing the porosity, releasing of l-ascorbic acid into solution increased due to the trapping of l-tyrosine in dense films. Gemili, Yemenicioğlu and Altınkaya [76] developed a cellulose acetate based antimicrobial food packaging material for controlled release of lysozyme. The highest soluble lysozyme activity, antimicrobial activity and release rate were obtained in the film prepared from 5% cellulose acetate solution including 1.5% lysozyme. It was observed that with increasing cellulose acetate concentration of the casting solution decreased the porosity of the films, as a result, reduced the release rate, maximum released lysozyme activities and the antimicrobial activities of the films. Potassium sorbate loaded cellulose acetate food packaging material was developed using supercritical phase inversion process. It was observed that the mean pore size of membrane increased with increasing the temperature and decreasing the pressure of the supercritical phase inversion process. In addition, the mean pore size decreased with increasing the cellulose acetate content of the film. The release pattern of potassium sorbate was consistent with the decrease of mean pore size of the membrane. Therefore, the release rates can be controlled by varying supercritical process condition and the cellulose acetate concentration of the film [79,80]. Chen et al. [81] prepared antimicrobial methylcellulose films containing chitosan and sodium benzoate or potassium sorbate to study the antimicrobial activity on *Penicillium notatum* and *Rhodotomla nibra*. The methylcellulose films with 2% antimicrobial agent showed a clear zone at the film/medium interface and the area around the disc. However, chitosan film containing 2% of antimicrobial agent was not showed a clear inhibitory zone around the film disc. The film composed of both methylcellulose and chitosan containing 4% antimicrobial agent was able to release the antimicrobial agents and showed clear inhibitory zone during incubation. Nisin grafted carboxylated cellulose nanofiber films were investigated for long term antimicrobial active food packaging. It was found that film showed an excellent antimicrobial activity on different Gram +ve bacteria with 3.5 log reduction of initial population [82].

## 4. Conclusions

Biopolymers offer developers the tremendous flexibility to design porous matrices to broaden its applications in different areas day by day. Different applications require a unique set of pore characteristics in the biopolymer matrix. In tissue engineering applications, appropriate scaffold pore sizes are required to facilitate seeding cells in the middle of the scaffold and for feeding the inner parts of the scaffolds by diffusion of nutrients to cells within the template and to the extra-cellular medium. Porous biopolymer matrices are popular candidates for drug delivery applications due to controllable pore sizes, high surface area, with narrow distribution and favorable surface properties. Pore size of the matrix plays a major role in drug diffusion kinetics. Chitosan, collagen, hyaluronic acid, alginate and elastin are attractive biopolymers as wound dressing materials due to the fungicidal properties, ability of providing extra cellular matrix, load-bearing and stretchable properties and absorbing large exudate volumes in wounds. In brewing and wine making industry, immobilization of microorganisms to biomaterials inhibits the toxic influence of produced ethanol on microorganisms and improves the process conditions as well as the properties of the product. Probiotic microorganisms encapsulated in biopolymer matrices can prevent contact with the extreme conditions in the gastrointestinal tract and hence providing more health benefits of probiotic products to the host. Antimicrobial packaging materials are developed with controlled releasing rates to overcome the food spoilages occur in surface of the foods. Preparation of biopolymer films containing antimicrobial agents is also a great attempt towards attaining sustainability in food packaging applications.

It can be seen that freeze-drying and supercritical fluid processing are widely used for the fabrication of porous scaffold for tissue engineering and wound healing matrices. This is due to the fact that these methods do not involve any organic solvent which may be harmful to interacting cells and this has ensured that no harmful residual solvents retained in the scaffolds after processing. While, the simple and versatile methods such as thermally induced phase separation and freeze drying process are widely used for fabrication of porous biomaterial for drug delivery and microbiological applications.

In summary, the advantages of biomaterials such as biodegradability, biocompatibility, easy availability, etc., have outweighed the limitations of synthetic materials for applications in fields such as biomedical and microbiology. It’s not compulsory for life changing developments to be high-tech devices with out-of-sight expectations. Sometimes the simplest contrivance can lead to a greater progress than expensive and advanced technologies. This is true for bio-based porous materials, too. The emerging properties of these materials lead to a pronounced increase in the range of use and the efficacy of biomaterials.

## Figures and Tables

**Table 1:** Optimal pore size for cell infiltration reported by previous researchers [25,26,27,28,29,30].

Cell types cultured	Optimal pore size (µm)	Reference
Hepatocytes	20 µm	[26]
Osteogenic cells	100–150 µm	[27]
Fibroblast	5–15 µm	[26]
Adult mammalian skin cells	20–125 µm	[28]
Smooth muscle cells	60–150 µm	[29]
Endothelial cells	<80 µm	[30]

**Table 2:** Summary of pore characteristics of biopolymer scaffolds prepared from different fabrication methods for culturing different types of cells [32,35,36,37,38].

Cell/tissue type	Biopolymer	Method of fabrication	Pore characteristics	Reference
Small-diameter blood vessels	Poly(l-lactic acid) (PLLA)	Thermally induced phase-separation	Porosity decreased from 95% to 90% with increasing the polymer concentration from 2.5% to 10%. Pore size decreased from 115–140 µm to 20–40 µm with decreasing the phase-separation temperature from −20 to −196 °C	[32]
Hepatocytes	Chitosan	Lyophilization	Porosity of 90% and mean pore size between 50–200 µm	[35]
Bone tissue	Hydroxyapatite and gelatin	Solvent-casting method combined with freeze drying	Open, interconnected porous structure with a pore size of 80–400 µm and porosity 70%	[36]
Human dermal fibroblasts	Chitosan	Freeze drying	Pore size between 40–140 µm, and average porosity about 93% ± 12.57%	[37]
Osteoblast	Poly(lactic-*co*-glycolic acid)/Hyaluronic acid/collagen	Supercritical CO_2_	Porosity of 88.9% and pore size of 205.7 µm	[38]

**Table 3:** Pore characteristics of different drug delivery systems prepared from various fabrication method [43,44,45,46,48,49,50,51].

Type of drug	Biopolymer	Method of fabrication	Pore characteristics	Reference
Doxycycline hyclate	Chitosan	Freeze drying	Well interconnected pores with diameter about 80–130 µm	[43]
Ampicillin and cytochrome C	Poly(lactic acid)	Robotic dispensing technique and room temperature ionic liquid	Pore size of 2.43 µm and microporosity of ~70%	[45]
Metoprolol succinate	Hydroxypropyl-methylcellulose and chitosan	Gas blowing	Pore size between 100–1000 µm and porosity of 47.11% ± 1.80%	[48]
Amoxicillin trihydrate	Chitosan	Freeze drying	Pore sizes were obtained from100 to 500 µm with increasing the crosslinking agent from 1:0.068 to 1:0.30 (molar ratio-chitosan: crosslinker)	[44]
Tetracycline hydrochloride	Hydroxyapatite/chitosan	Freeze drying	Pore diameter 45 ± 17 µm.	[46]
Rosiglitazone maleate	Chitosan/poly(vinyl alcohol)	Gas foaming	Superporous hydrogel with capillary porous structures. Porosity increased from 38.3 ± 2.2 to 88.2 ± 2.1 with increasing the amount of glyoxal (crosslinker)	[49]
Ranitidine	Carboxymethylcellulose hydrogel	Gas foaming	Porosity decreased from 69.30 ± 4.36 to 42.38 ± 2.68 with the addition of sodium carboxymethyl cellulose	[50]
Curcumin	Nanocellulose reinforced chitosan hydrogel	Gas foaming	Highly interconnected pores with pore sizes >100 µm	[51]
